# Translocator protein deficiency blocks the ferroptosis of malignant peripheral nerve sheath tumors through glutathione peroxidase 4

**DOI:** 10.3389/fncel.2025.1624817

**Published:** 2025-08-06

**Authors:** Xiaoli Zhang, Zhuonan Pu, Chun Ran, Xingnan Zhang, Chao Guo, Yuxuan Deng, Jinqiu Liu, Yingdan Chen, Jie Feng, Song Liu

**Affiliations:** ^1^Department of Injury and Repair, Beijing Neurosurgical Institute, Capital Medical University, Beijing, China; ^2^Department of Gastrointestinal Surgery, Peking University Shenzhen Hospital, Shenzhen, China; ^3^China Ordnance Society, Beijing, China; ^4^U1195, Inserm et Universite Paris-Saclay, Le Kremlin-Bicetre, France

**Keywords:** malignant peripheral nerve sheath tumor, translocator protein, ferroptosis, lipid peroxidation, glutathione peroxidase 4

## Abstract

**Background:**

Malignant peripheral nerve sheath tumor (MPNST) is an aggressive soft tissue sarcoma characterized by high recurrence and poor prognosis, necessitating the search for novel therapeutic targets and strategies. This study investigated the expression of mitochondrial translocator protein (TSPO) in MPNST and its role in regulating ferroptosis.

**Methods:**

TSPO expression was analyzed in adjacent non-tumor tissues, benign neurofibromas, and malignant tissues using real-time PCR, western blotting, immunohistochemistry staining. Expression levels of classic ferroptosis markers, including AKR1C1 and FTH1 were assessed. Ferroptosis was evaluated by measuring cell viability, ferroptosis marker levels, and intracellular Fe^2+^ and reactive oxygen species (ROS) levels. Oxidized phospholipid profiles of wild-types and *TSPO* knockdown MPNST cells were determined using liquid chromatography-mass spectrometry. The potential role of GPX4 in mediating TSPO’s effect on ferroptosis was investigated *in vitro*.

**Results:**

Compared with adjacent non-tumor tissues and benign neurofibromas, TSPO expression was significantly lower in MPNST specimens. Notably, TSPO expression positively correlated with the classic ferroptosis markers AKR1C1 and FTH1. TSPO-knockdown MPNST cells exhibited significant resistance to ferroptotic cell death. Additionally, biochemical characterization indicated that TSPO deficiency decreased intracellular Fe^2+^ and ROS. Furthermore, oxidized phospholipids were remarkably reduced in TSPO-knockdown cells. TSPO enriches cellular oxidized phospholipids by downregulating GPX4-GSH antioxidant pathway. Furthermore, GPX4 is elevated in malignant tumors compared to benign specimens and negatively correlated with TSPO expression in clinical tumor specimens.

**Conclusion:**

Our findings revealed that TSPO deficiency inhibited ferroptosis in MPNST cells by upregulating GPX4 antioxidant pathway, suggesting that mitochondrial TSPO-GPX4-ferroptosis axis may be a promising therapeutic target for improving the outcomes of patients with MPNST.

## Introduction

Malignant peripheral nerve sheath tumor (MPNST) is a highly aggressive soft tissue sarcoma associated with peripheral nerves and is characterized by a high recurrence rate and poor prognosis (Xiao et al., 2025). MPNSTs typically arise from benign neurofibromas (NFs) and account for 2–10% of soft tissue sarcomas annually (Siegel et al., 2024). Currently, the most effective treatment for localized MPNST involves surgical resection accompanied by chemotherapy and radiation therapy (Clark et al., 2005; Dunn et al., 2013). For patients with unresectable tumors or metastatic disease, chemotherapy or targeted radiotherapy remain the primary treatment options, though they extend life expectancy by 1–2 years (van Noesel et al., 2019). The overall prognosis remains poor, with a five-year overall survival rate of just 47.2% (Kolberg et al., 2013). The limited effectiveness of these therapies is primarily due to invasive tumor growth, high propensity to metastasis, and resistance to treatment. Therefore, there is an urgent need to explore novel targets and therapeutic strategies for the treatment of MPNST.

Ferroptosis, a programmed cell death form, is initiated by iron overload, and ROS accumulation (Lei et al., 2022; Zhang et al., 2023). Iron accumulation, especially ferrous iron (Fe^2+^), can give rise to ROS through the Fenton reaction, ultimately initiating ferroptosis (Dixon et al., 2012; Zhang et al., 2022). Notably, lipid peroxidation mediated by excessive ROS is a prerequisite of ferroptosis. Polyunsaturated fatty acids are readily abstracted and susceptible to lipid peroxidation. Generally, arachidonic acid (AA)- and adrenic acid (AdA)-containing membrane phospholipids are the prime substrates for lipid peroxidation (Zhang et al., 2023). To counteract lipid peroxidation, cells have developed efficient antioxidant defense systems. Glutathione peroxidase 4 (GPX4) is a critical lipid ROS scavenger and can catalyze lipid peroxides into non-hazardous lipid alcohols to protect cells against ferroptotic death (Tang et al., 2021; Yang et al., 2014).

Translocator protein (TSPO) is identified as a highly conserved protein residing on the outer mitochondrial membrane, and involved in diverse cellular functions, including iron homeostasis, oxidative stress regulation, and metabolism (Li et al., 2024). Dysregulated TSPO expression has been implicated in various cancer types, such as breast cancer (Galiègue et al., 2004), colorectal cancer (Xie et al., 2021), and glioblastoma (Menevse et al., 2023). Our previous study revealed that TSPO is downregulated in MPNST specimens and regulates MPNST progression and development by targeting cyclin-dependent kinase 1 (Zhang et al., 2024). Additionally, TSPO has been reported to play a regulatory role in lipid metabolism, and its inhibition leads to the generation of various free fatty acids in tanycytes (Kim et al., 2020). Furthermore, TSPO has been shown to regulate ferroptosis in hepatocellular carcinoma cells via P62-NRF2 pathway (Zhang et al., 2023). However, the role of TSPO in the regulation of lipid peroxidation and ferroptosis in MPNST remains unclear. This study aimed to investigate the role of TSPO in regulating the ferroptotic sensitivity of MPNST cells. Moreover, we explored the underlying mechanism of TSPO in the regulation of lipid peroxidation and ferroptosis. Our findings broaden the understanding of MPNSTs and indicate that TSPO may be a promising novel therapeutic target for MPNST therapy.

## Materials and methods

### Human clinical samples

Clinical tissues (Supplementary Table S1) were collected from patients with NF (18 cases) and MPNST (12 cases) at the Department of Neurosurgery at Beijing Tiantan Hospital Affiliated with Capital Medical University (Beijing, China), between 2019 and 2023. For four of the MPNST patients, adjacent non-tumor tissues (referred to as peritumor) were also obtained. The inclusion criteria were tumor specimens resected from patients initially diagnosed with NF or MPNST. The exclusion criteria were patients initially diagnosed with non-NF or non-MPNST conditions, or those with recurrent tumors. Human tissue samples were gathered from every patient who had offered written informed consent. The standardized procedures for human samples collection and utilization were approved by the Internal Review Board of Beijing Tiantan Hospital Affiliated with Capital Medical University (KYSQ 2025-334-01). Clinical trial number: not applicable.

### Cell culture

The human plexiform neurofibroma cell line ipNF05.5 (RRID: CVCL_UI71) and MPNST cell line sNF96.2 (RRID: CVCL_K281), purchased from the American Type Culture Collection (Manassas, VA, United States), were cultured in DMEM (Gibco) and RPMI 1640 (Gibco) medium respectively, supplemented with 1% penicillin and streptomycin and 10% foetal bovine serum (Gibco). The cells were cultured at 37°C in a humidified, 5% CO_2_ environment. Both human cell lines have been authenticated using STR (or SNP) profiling, and all experiments were performed with mycoplasma-free cells.

### Immunohistochemistry (IHC) analysis

Tissue samples embedded in paraffin were precisely sectioned into 4 mm sections. These sections were then meticulously dewaxed and hydrated. Afterwards, they were incubated with following primary antibodies at 4°C overnight. TSPO (ABclonal, A4881, 1:200), FTH1 (Abcam, ab287968, 1:100), and AKR1C1 (Abcam, ab192785, 7.5 μg/mL) antibodies. The signals were visualized and images were analyzed as described previously (Zhang et al., 2021).

### Reverse transcription-quantitative polymerase chain reaction (RT-qPCR)

The total RNA was isolated from tissues cryopreserved in liquid nitrogen or cells and reverse transcribed into cDNA as described previously (Zhang et al., 2022). The results underwent normalization against the expression of glyceraldehyde-3-phosphate dehydrogenase. The sequences of the GAPDH, TSPO, AKR1C1, FTH1, GPX4, TP53, P21 and NRF2 primers used in this study are presented in Supplementary Table S2.

### Western blotting

Proteins were isolated from tumor tissues cryopreserved in liquid nitrogen or cells and protein concentrations were quantified as described previously (Zhang et al., 2021). 100 μg lysate was subjected to separation on a 10% sodium dodecyl sulfate-polyacrylamide gel electrophoresis gel. Following electrophoretic separation, the proteins were transferred onto polyvinylidene difluoride membranes. Subsequently, the membranes were incubated with following primary antibodies during an overnight incubation at 4°C. TSPO (ABclonal, A4881, 1:1000), GPX4 (Proteintech, 67763-1-Ig, 1:1000) and β-actin (Invitrogen, A1978, 1:10000) antibodies. Subsequently, the membranes were incubated with a peroxidase-conjugated secondary antibody (Cell Signaling Technology, 1:10000) for 1.5 h. The signal was visualized through the application of enhanced chemiluminescence reagent (Millipore).

### Ferrous iron staining

We used an Fe^2+^ indicator (FeRhoNox-1, MKbio, MX4558) to detect ferrous iron in tumor cells as described previously (Zhang et al., 2022). Cells were incubated for 1 h at 37°C with FeRhoNox-1. The cells were then harvested by trypsinization, and the level of ferrous iron was determined by imaging using a confocal microscope or by flow cytometry analysis.

### Oxidized phospholipids analysis

The extraction of lipids from approximately one million cells was conducted utilizing an adapted Bligh and Dyer’s method as described previously (Song et al., 2020). The lipidomic analysis was carried out at LipidALL Technologies by utilizing an ExionLC-AD-Sciex QTRAP 6500 PLUS mass spectrometer as previously reported (Lam et al., 2021). The separation of distinct lipid classes within oxidized phospholipids was accomplished through normal phase-high performance liquid chromatography. A TUP-HB silica column (i.d. 150 × 2.1 mm, 3 μm) was employed for this separation process. The chromatographic conditions were as follows: mobile phase A consisted of a mixture of chloroform, methanol, and ammonium hydroxide in a ratio of 89.5:10:0.5, and mobile phase B comprised chloroform, methanol, ammonium hydroxide, and water in a ratio of 55:39:0.5:5.5. Multiple reaction monitoring transitions were configured to facilitate the comparative analysis of diverse oxidized phospholipids. Quantification of individual lipid species was achieved by referencing the responses against spiked internal standards.

### Reduced glutathione assay

We used a reduced glutathione assay kit to measure the reduced GSH as described previously (Zhang et al., 2021). The cells were collected, washed twice with ice-cold PBS, lysed by freezing and thawing twice in liquid nitrogen at 37°C, and centrifuged. The supernatant was collected and used for the assays. GSH standard solutions and samples were loaded into 96-well plates. Next, a buffer solution was added to each well, and the plate was incubated at 25°C for 5 min. NADPH solution was then added to each well and the plate was incubated at 25°C for 30 min, and the absorbance was read at 412 nm using microplate reader. The GSH concentrations were normalized to the total protein levels in each sample.

### Statistical analysis

Unless explicitly indicated otherwise, all experimental procedures were independently replicated three times. Statistical analyses were conducted utilizing the GraphPad Prism software (version 8.0), a widely recognized and validated tool in the field of data analysis. The experimental data are presented as the mean ± standard deviation (SD). For the comparison of two groups, the Student’s *t*-test for unpaired data was applied. Comparisons among more than two groups were performed using ANOVA and Tukey’s test. For all statistical tests conducted in this study, a significance level of *p* ≤ 0.05 was adopted, and results with *p* values below this threshold were denoted with an asterisk (*p < 0.05) to indicate statistical significance.

## Results

### TSPO is lowly expressed in MPNST specimens

**Figure 1:**
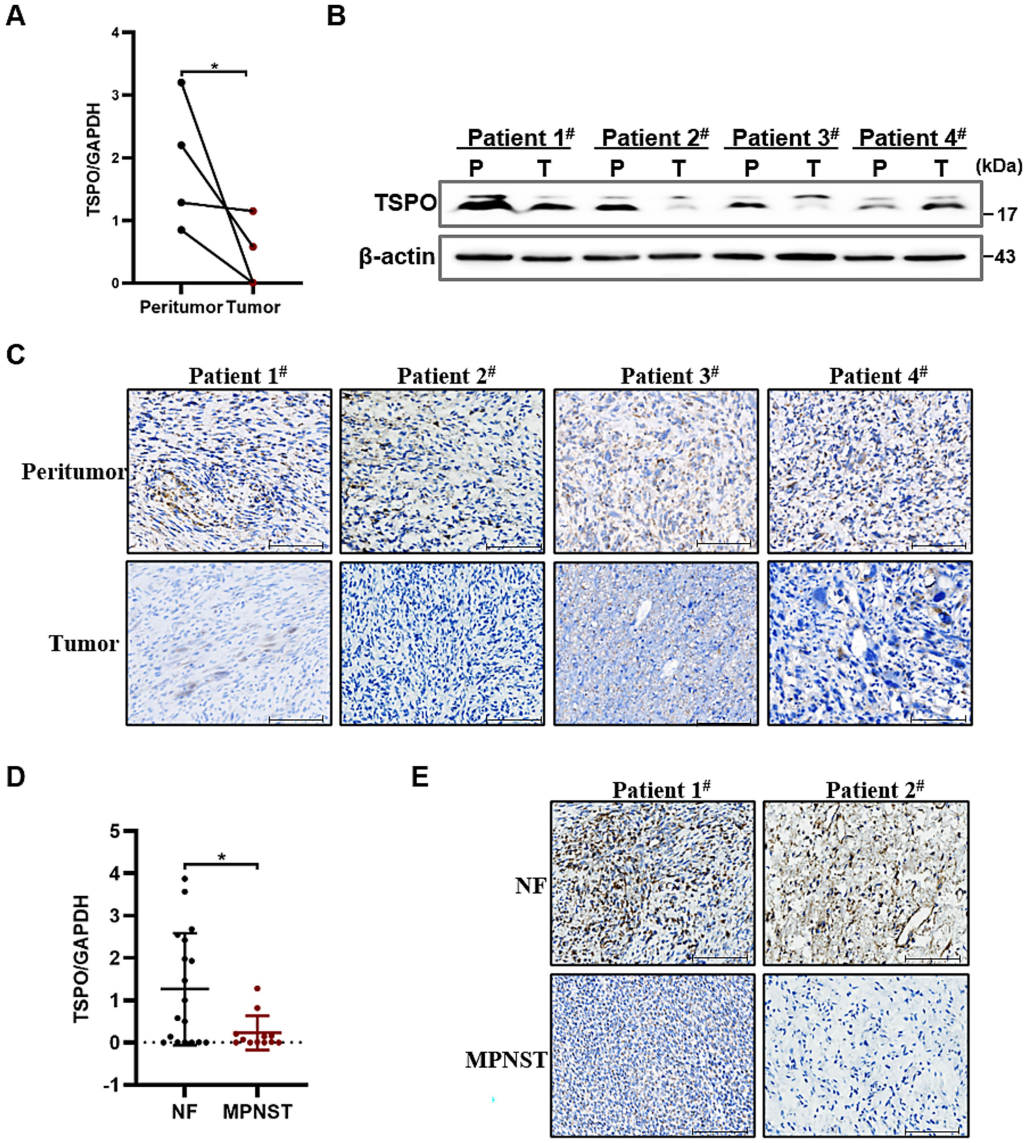
TSPO is lowly expressed in MPNST specimens. **(A)** Quantitative analysis of TSPO mRNA expression in paired MPNST tissues and their corresponding adjacent non-tumor tissues (*n* = 4). **(B)** TSPO protein expression in paired MPNST tissues and their corresponding adjacent non-tumor tissues (*n* = 4). **(C)** TSPO of biopsies from 4 paired MPNST tissues and their corresponding adjacent non-tumor tissues by IHC staining (scale: 100 μm). **(D)** TSPO mRNA expression in benign NF tissues (*n* = 18) and malignant MPNST tissues (*n* = 12). **(E)** TSPO of biopsies from benign NF tissues and malignant MPNST tissues by immunohistochemistry staining (scale: 100 μm). Statistical significance was determined using one-way ANOVA with Tukey’s test. *p < 0.05.

To explore TSPO expression in tumors, we analyzed data from the TIMER database and found that TSPO mRNA expression varied depending on tumor type, with notably high expression in liver cancer and comparatively low expression in lung cancer (Supplementary Figure S1A). We performed RT-qPCR and western blotting on four pairs of MPNST tissue samples, with each pair originating from a single patient. The results showed that TSPO protein expression in MPNST tissues was lower than that in the adjacent non-tumor tissues (Figures 1A,B and Supplementary Figure S1B). We further validated the decreased expression of TSPO in MPNST clinical specimens using IHC staining (Figure 1C). Moreover, we extracted RNA from 18 benign and 12 malignant patient tissues and detected low TSPO expression in MPNST tissues using RT–qPCR (Figure 1D). IHC staining also confirmed that TSPO was expressed at low levels in malignant tumor samples (Figure 1E). These findings indicate that TSPO is downregulated in malignant tumor specimens.

### TSPO is positively correlated with ferroptosis markers

**Figure 2:**
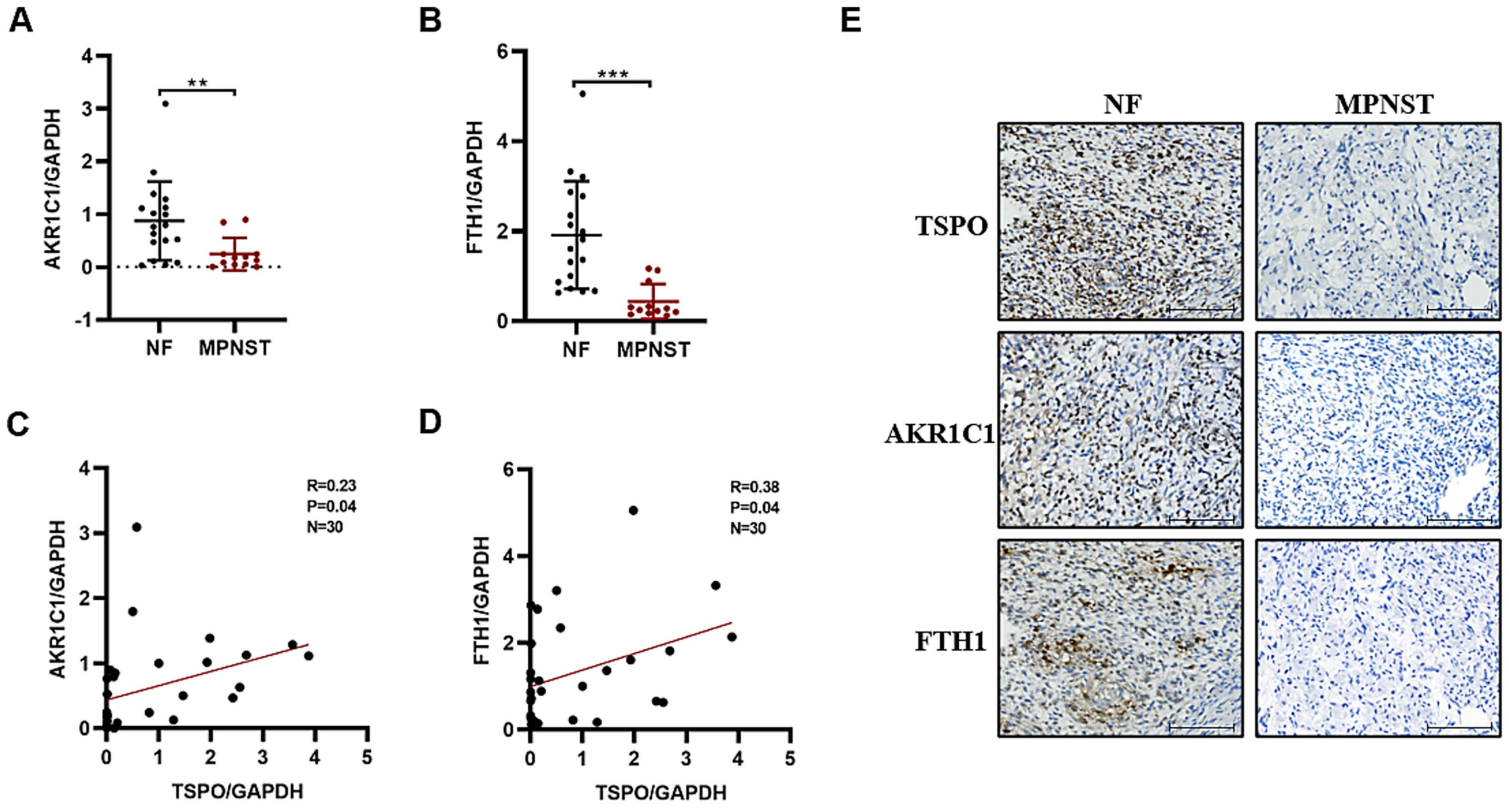
TSPO is positively correlated with ferroptosis markers in tumor specimens. **(A)** AKR1C1 mRNA expression in benign NF tissues (*n* = 18) and malignant MPNST tissues (*n* = 12) evaluated by RT-qPCR. **(B)** FTH1 mRNA expression in benign NF tissues (*n* = 18) and malignant MPNST tissues (*n* = 12) evaluated by RT-qPCR. **(C)** The correlation analysis was conducted for TSPO/AKR1C1 in tumor specimens. **(D)** The correlation analysis was conducted for TSPO/FTH1 in tumor specimens. **(E)** Immunohistochemical staining for TSPO, AKR1C1 and FTH1 protein expressions in NF and malignant MPNST tissues (scale: 100 μm). Statistical significance was determined using one-way ANOVA with Tukey’s test. **p < 0.01, ***p < 0.001.

To investigate the relationship between TSPO and ferroptosis in MPNST, we used RT-qPCR to detect the differential mRNA expression levels of two classic ferroptosis biomarkers, AKR1C1 and FTH1, in tissues from patients with benign and malignant tumors. Compared with benign specimens, ferroptosis biomarkers were downregulated in malignant tumors (Figures 2A,B), which was consistent with the anti-death characteristics of tumor cells. Notably, TSPO expression positively correlated with ferroptosis markers (Figures 2C,D). IHC staining showed that malignant tumors with high TSPO expression had significantly higher AKR1C1 and FTH1 expression levels. Conversely, in tumor tissues with low TSPO expression, AKR1C1 and FTH1 are similarly found to have low expression (Figure 2E). These findings indicate that TSPO may play a vital role in regulating ferroptosis in MPNST.

### TSPO deficiency MPNST cells are highly resistant to ferroptosis

**Figure 3:**
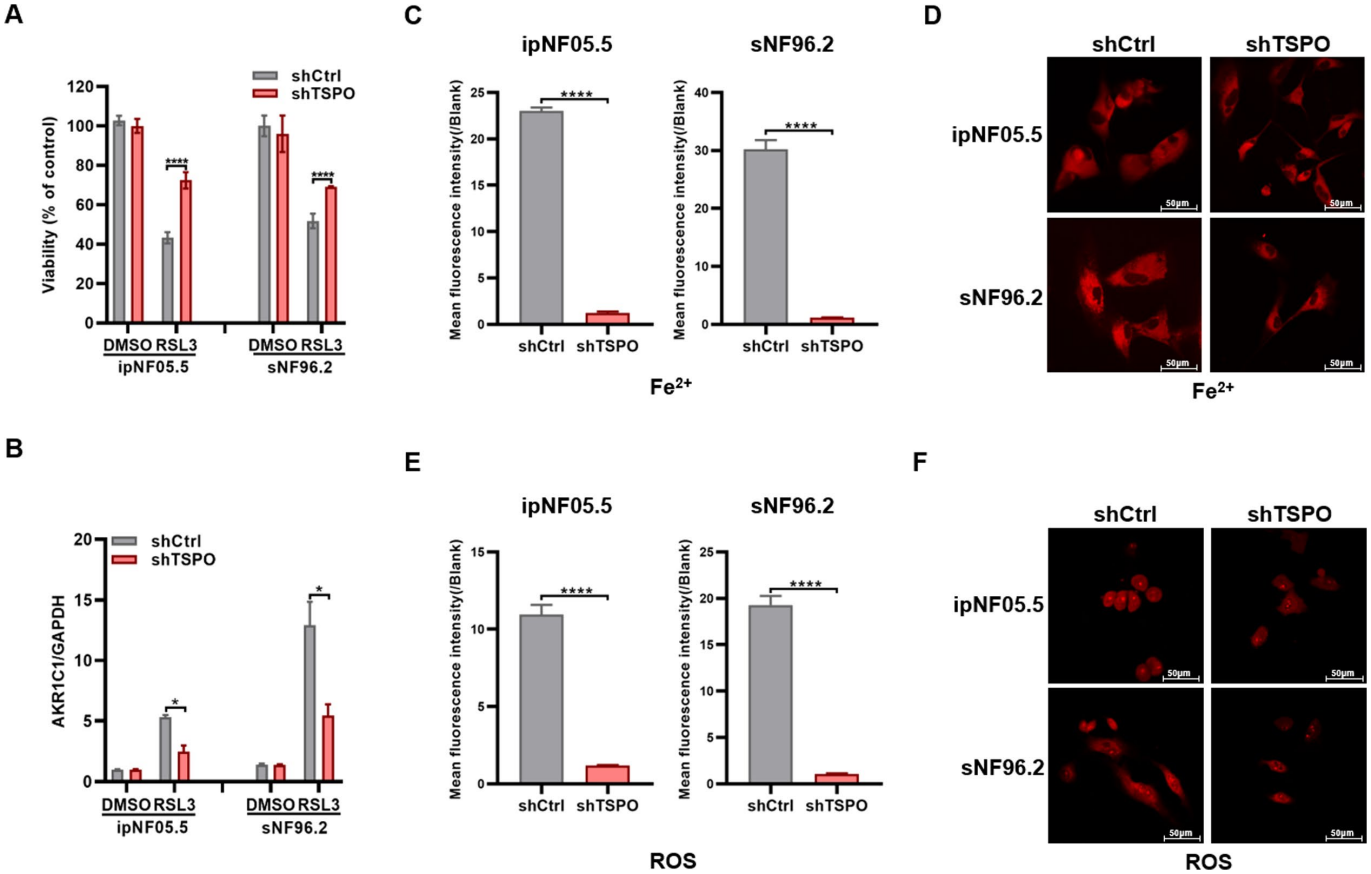
TSPO deficiency cells are resistant to ferroptosis. **(A)** Trypan blue stain assessment of human ipNF05.5 and sNF96.2 cell death. **(B)** AKR1C1 mRNA expression evaluated by RT-qPCR. **(C,D)** Cytosolic Fe^2+^ level was conducted utilizing flow cytometry **(C)** and confocal microscopy (**D**, scale: 50 μm). **(E,F)** Cytosolic ROS level was conducted utilizing flow cytometry **(E)** and confocal microscopy (**F**, scale: 50 μm). Statistical significance was determined using one-way ANOVA with Tukey’s test. *p < 0.05, ****p < 0.0001.

To address the putative role of TSPO in the ferroptosis, we first established ferroptosis models using a ferroptosis inducer (1S,3R-RSL3) in the MPNST cell line sNF96.2. In alignment with prior reports (Zhang et al., 2021), the cell death triggered by RSL3 was fully reversed by Fer-1, a specific inhibitor of ferroptosis, but not by Z-VAD-FMK (an inhibitor of apoptosis) (Supplementary Figure S2A). The MPNST cells sNF96.2 were more resistant to ferroptosis induced by various concentrations of RSL3 than neurofibroma cells ipNF05.5 (Supplementary Figure S2B). The effect of TSPO knockdown on ferroptosis was assessed by trypan blue staining. As shown in Figure 3A, TSPO deficient tumor cells are insensitive to ferroptosis. The inhibition of ferroptosis by TSPO deficiency was supported by the measurement of ferroptosis markers using RT-qPCR (Figure 3B, and Supplementary Figure S2C). In addition, TSPO knockdown decreased the levels of intracellular Fe^2+^ and ROS, as measured using FeRhoNox-1 and DCFH-DA, respectively (Figures 3C–F). These results suggest that TSPO deficiency attenuates ferroptosis in human MPNST cells.

### TSPO knockdown decreases the levels of various oxidized phospholipids

**Figure 4:**
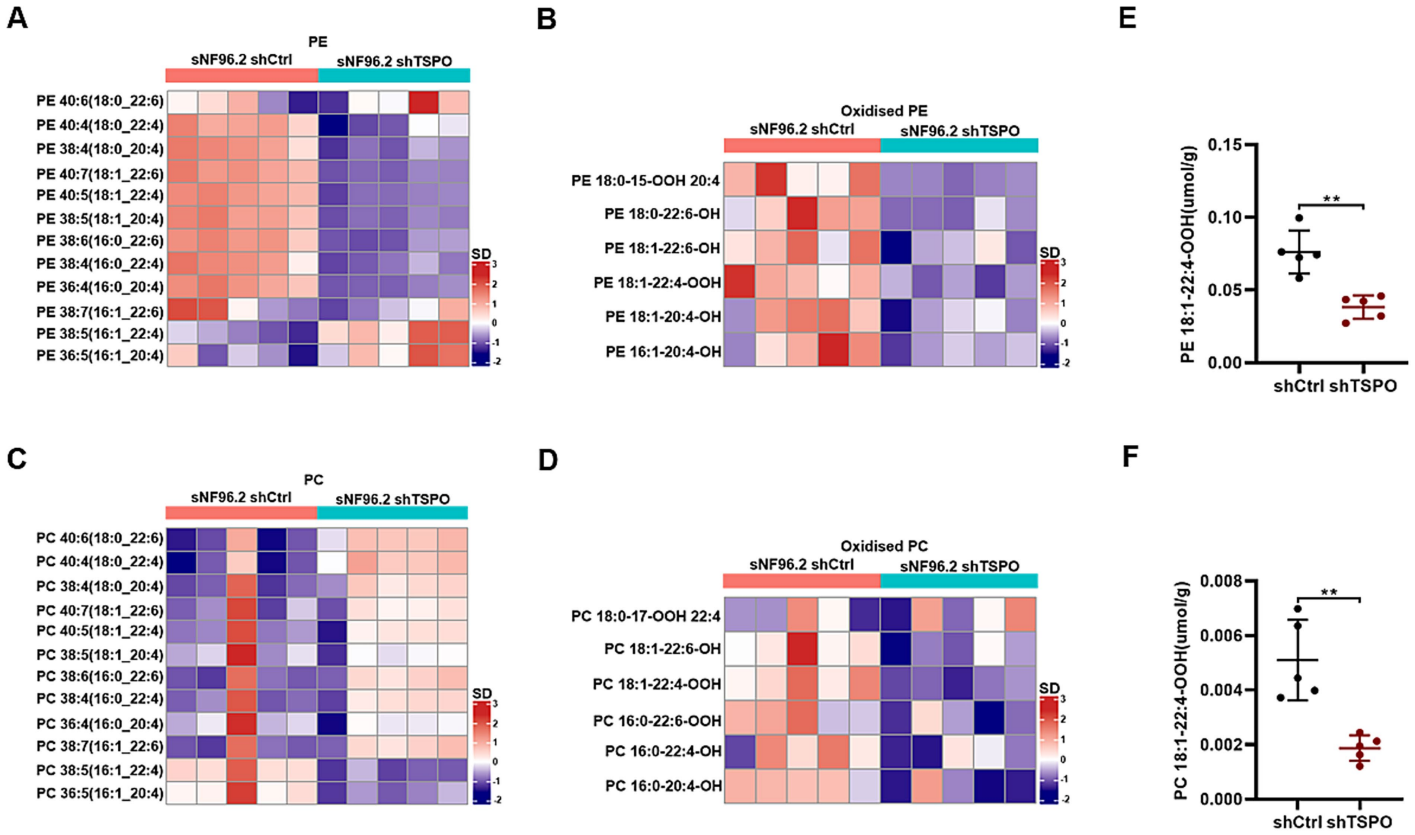
TSPO knockdown decreases various oxidized phospholipids. **(A,B)** Quantitative assessment of PE **(A)** and oxidized PE **(B)** molecular species in WT and TSPO knockdown sNF96.2 cells. **(C,D)** Quantitative assessment of PC **(C)** and oxidized PC **(D)** molecular species in WT and TSPO knockdown sNF96.2 cells. **(E)** Quantitative assessment of hydroperoxy-PE molecular species (18:1/22:4). **(F)** Quantitative assessment of hydroperoxy-PC molecular species (18:1/22:4). Statistical significance was determined using one-way ANOVA with Tukey’s test. **p < 0.01.

Ferroptosis is fundamentally orchestrated by the peroxidation of phospholipids bearing polyunsaturated fatty acids, leading to the deleterious accumulation of lipid peroxides on cellular membranes and eventually resulting in membrane disruption (Lei et al., 2022). To understand how loss of TSPO provides protective effects, we analyzed the difference in oxidized phospholipids in wild-type (WT) and TSPO knockdown sNF96.2 cells by liquid chromatography-mass spectrometry. TSPO-knockdown cells exhibited distinctive oxidized phospholipid (PL) profiles that differed from those of WT cells (Supplementary Figure S3A). Phosphatidylethanolamine (PE) and oxidized PE levels significantly decreased in TSPO-knockdown cells (Figures 4A,B, and Supplementary Figure S3B). Although TSPO deficiency increased phosphatidylcholine (PC) levels, it reduced oxidized PC levels, which play a crucial role in ferroptosis (Figures 4C,D, and Supplementary Figure S3B). AA (20:4) and AdA (22:4), which are present in phospholipids, are the preferred substrates for oxidation (Doll et al., 2017). A detailed analysis of the potentially oxidized phospholipids was performed. We found that the respective oxidized species were more abundant in the WT cells, especially the hydroperoxy-PE (18:1/22:4) and hydroperoxy-PC (18:1/22:4) molecular species (Figures 4E,F). Overall, these findings indicate that TSPO deficiency is accompanied by a substantial decrease in oxidized phospholipids, which are used as substrates to trigger ferroptosis.

### TSPO reduction inhibits ferroptosis through GPX4-GSH antioxidant system

**Figure 5:**
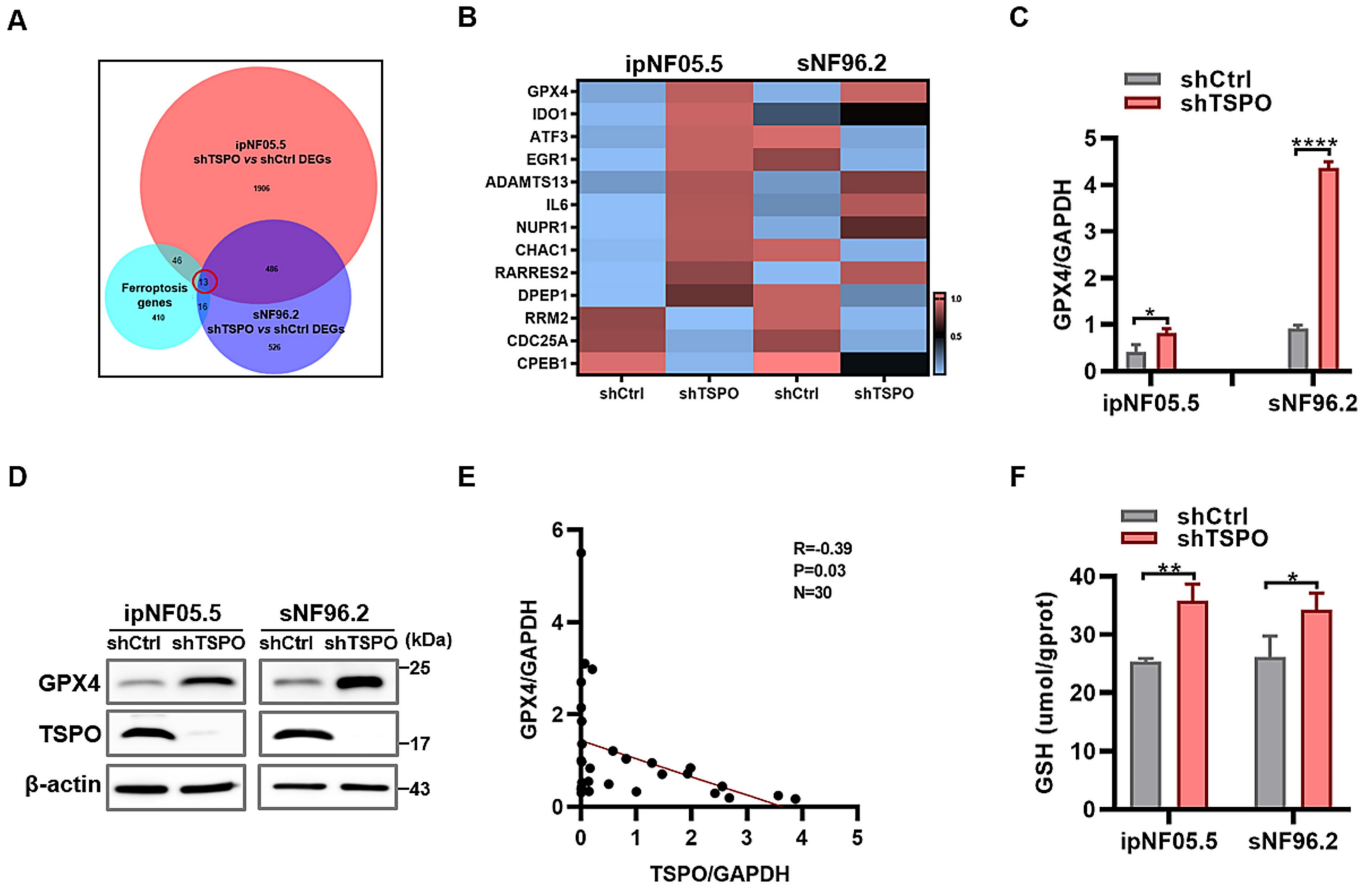
TSPO reduction inhibits ferroptosis through GPX4-GSH antioxidant system. **(A)** Venn diagram showing the intersection of the differentially expressed genes in ipNF05.5 and sNF96.2 cells (shTSPO vs. shCtrl) with ferroptosis-related genes from the FerrDB database. **(B)** Heatmap showing the intersected genes. **(C)** GPX4 mRNA expression evaluated by RT-qPCR. **(D)** GPX4 protein expression evaluated by Western blot. **(E)** The correlation analysis was conducted for TSPO/GPX4 in tumor specimens. **(F)** GSH level was measured in WT and TSPO knockdown ipNF05.5 and sNF96.2 cells. Statistical significance was determined using one-way ANOVA with Tukey’s test. *p < 0.05, **p < 0.01, ****p < 0.0001.

To further elucidate the mechanisms by which TSPO deficiency inhibits ferroptosis, transcriptome analysis was performed in WT and TSPO-knockdown ipNF05.5 and sNF96.2 cells, with three replicates for each group. We identified 13 genes by intersecting the differentially expressed genes in each group (shTSPO vs. shCtrl) with 485 ferroptosis-related genes from the FerrDB database (Figure 5A). Among these, the GPX4 mRNA levels were significantly increased in the TSPO-knockdown groups (Figure 5B). GPX4 is the key antioxidant and ferroptosis defence system that convertes PL hydroperoxides to PL alcohols to protect cells against ferroptotic death. We further validated the marked upregulated in TSPO-knockdown cells by using RT-qPCR and western blotting (Figures 5C,D). Furthermore, GPX4 is elevated in malignant tumors compared to benign specimens and is negatively correlated with TSPO expression in tumor specimens (Figure 5E and Supplementary Figure S4). TSPO deficiency increased the levels of reduced glutathione (GSH), a cofactor used by GPX4 (Figure 5F). These observations suggest that TSPO deficiency blocks the ferroptotic death of MPNST cells by upregulating the GPX4 antioxidant system and subsequently decreasing lipid peroxidation.

## Discussion

TSPO is localized on the outer membrane of the mitochondria and plays a crucial role in maintaining cellular function. TSPO regulates metabolism, oxidative stress, and apoptosis (Pan et al., 2023). Recent studies have demonstrated that TSPO plays an important role in carcinogenesis and the expression and function of TSPO varied inconsistently across different types of tumors. TSPO is upregulated in liver (Zhang et al., 2023), breast (Hardwick et al., 1999), and pancreatic cancers (Zhang et al., 2021), while significantly downregulated in colon and lung cancers (Bhoola et al., 2018), resulting in differential effects on the biological phenotype of tumor cells. Notably, TSPO plays dual roles in promoting and inhibiting gliomas (Fu et al., 2020). In a previous study (Zhang et al., 2024), we carried out transcriptome sequencing on three pairs of benign and malignant tissues and RT-qPCR on four benign and eight malignant patient tissues, all of which showed low TSPO expression in the MPNST. In our study, we added the adjacent non-tumor tissues and expanded the sample size to include four normal and malignant tissue pairs (each from a single patient), and an additional 18 benign and 12 malignant patient tissues. The expression level of TSPO in tumor tissues was lower compared with the adjacent non-tumor tissues. Consistent with our previous studies, TSPO expression was lower in MPNST than in benign tumors. Therefore, the above findings further confirm the low expression of TSPO in MPNST and suggest that TSPO may play a tumor-suppressive role in the tumorigenesis and development of MPNST.

Ferroptosis has recently attracted substantial scholarly interest within the cancer research community due to its therapeutic potential (Niu et al., 2025). Mitochondria play a pivotal role in oxidative metabolism and have been implicated in the mechanisms underlying ferroptosis (Xiong et al., 2025). Currently, there is limited research on TSPO and ferroptosis in tumors. A recent study has reported that mitochondrial TSPO is highly expressed and inhibits ferroptosis in hepatocellular carcinoma cells by enhancing the Nrf2-dependent antioxidant defense system (Zhang et al., 2023). Mechanistically, TSPO directly interacts with P62 and leads to the accumulation of P62 which prevents proteasomal degradation of Nrf2. Conversely, the mitochondrial TSPO was downregulated in MPNST and promoted malignant cell ferroptosis. In NF and MPNST clinical specimens, we observed a significant positive correlation between the expression levels of TSPO and the levels of the ferroptosis markers AKR1C1 and FTH1. *In vitro* cellular experiments showed that TSPO knockdown reduced the sensitivity of MPNST cells to ferroptosis, accompanied by significant decreases in intracellular ferrous ion, ROS and peroxidized lipids levels. The different results between hepatocellular carcinoma and MPNST may be related to the different cancer types and genetic background, which require further in-depth study.

Recently, there has been a steep increase in research on ferroptosis in cancer, partly because of its potential as a target for therapeutic interventions. The involvement of ferroptosis in the activity of the tumor suppressor p53 underscores its role as a natural defense mechanism against cancer progression (Jiang et al., 2015). A distinctive tumor microenvironment with high ROS levels may render some cancer cells intrinsically susceptible to ferroptosis (Lei et al., 2022). However, the strong anti-lipid oxidation system of cancer cells enables them to cope with ROS-induced oxidative damage and survive in harsh environment (Zhang et al., 2023). Our results demonstrated that the expression profiles of GPX4 at both the protein and mRNA levels, as well as the GSH level, were higher in TSPO-knockdown cells, and that the mRNA level of TSPO was negatively correlated with GPX4 in clinical specimens. These results suggest that TSPO inhibits the antioxidant system of MPNST cells, thereby promoting ferroptosis.

Despite the significant findings, this study had some limitations. Our study primarily focused on *in vitro* models of MPNST and *in vivo* data from clinical samples, and the lack of *in vivo* animal experimental data limits the translational relevance of our findings. Establishing xenograft mouse models is essential for validating the therapeutic potential of targeting TSPO or GPX4 *in vivo*. Moreover, as a transmembrane protein rather than a transcription factor, the mechanism by which TSPO regulates the expression of GPX4 remains unclear. To delve deeper into the regulatory mechanisms governing GPX4 expression at the transcriptional level, we focused our investigation on the transcription factors involved. Among these factors, P53 and NRF2 emerged as being closely associated with TSPO. Prior research has extensively documented that p53 plays a pivotal role in sensitizing cells to ferroptosis by curbing the enzymatic activity or downregulating the expression of GPX4 (Jiang et al., 2024; Liu and Gu, 2022). In our study, we used RT-qPCR to assess the mRNA expression levels of P53 and its downstream gene P21 in WT and TSPO-knockdown ipNF05.5 and sNF96.2 cells. The P53 and P21 were downregulated in shTSPO cells vs. shCtrl cells, which is consistent with our previous research (Zhang et al., 2024). Furthermore, we conducted RT-qPCR analysis to evaluate the mRNA expression levels of NRF2 in the same cell lines. However, no discernible differences were observed between WT and TSPO-knockdown cells (Supplementary Figure S5). These results imply that TSPO may exert its influence on GPX4 expression through the regulation of P53. Nonetheless, the precise molecular mechanisms underlying this interaction warrant further elucidation.

## Conclusion

In this study, we detected TSPO expression in NF and MPNST clinical samples and investigated the effects of TSPO on ferroptosis in MPNST cells, as well as its underlying mechanisms. The findings of this study showed that TSPO is expressed at low levels in tumor tissues, and TSPO deficiency leads to a reduction in lipid accumulation by upregulating GPX4, thereby inhibiting ferroptotic cell death. Additionally, these findings provide a theoretical foundation for forthcoming studies aimed at developing more effective and personalized treatments for patients with MPNSTs.

## Data Availability

The datasets presented in this study can be found in online repositories. The names of the repository/repositories and accession number(s) can be found below: https://www.ncbi.nlm.nih.gov/geo/, GSE270880.
