# Supplementary material for: Translocator protein deficiency blocks the ferroptosis of malignant peripheral nerve sheath tumors through glutathione peroxidase 4
Source: Front Cell Neurosci. 2025 Aug 6;19:1624817. doi: 10.3389/fncel.2025.1624817 (PMC12364865; doi:10.3389/fncel.2025.1624817)
Supplement: Supplementary file 1 [file Supplementary_file_1.pdf]

Translocator protein deficiency blocks the ferroptosis of malignant peripheral nerve sheath tumors through glutathione peroxidase 4

Xiaoli Zhang [1,2#], Zhuonan Pu [1#], Chun Ran [3*], Xingnan Zhang [1], Chao Guo [1], Yuxuan Deng [1], Jinqiu Liu [1], Yingdan Chen [1], Jie Feng [1*], Song Liu [1,4*]

Figure S1

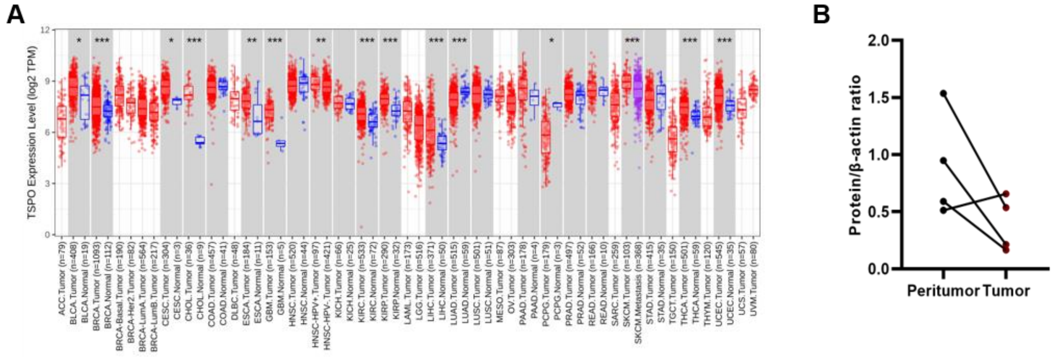

Figure S1. TSPO expression in different tumor tissues and adjacent non-tumor and MPNST tissues.

(**A**) TSPO mRNA expression in different tumor tissues based on the TIMER database.

(**B**) TSPO densitometry relative to β-actin related to Figure 1B.

Figure S2

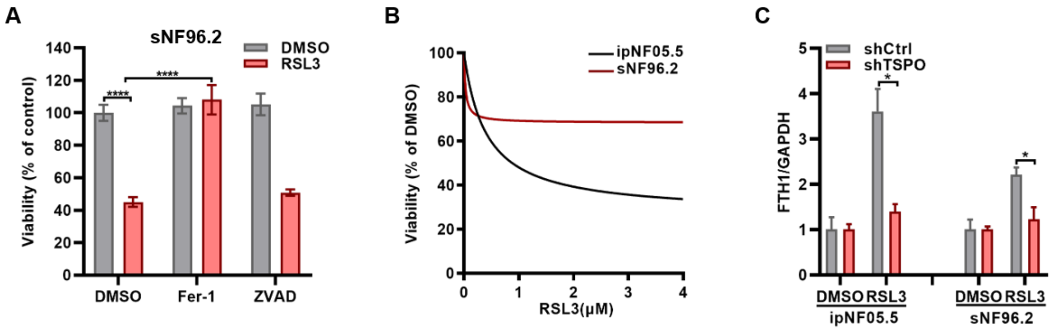

Figure S2. Establishment of ferroptosis models, comparison of ferroptosis sensitivity in ipNF05.5 and sNF96.2 cells, and TSPO modulates FTH1 in a ferroptotic context.

(**A**) Trypan blue stain assessment of human sNF96.2 cell death 24 h ± 1 μM RSL3, 2μM ferrostatin-1 (Fer-1) and Z-VAD-FMK (ZVAD). (**B**) Survival curves representing the viability of human ipNF05.5 and sNF96.2 cells upon treatment with the indicated RSL3 concentrations for 12 h. Each data point is expressed as the percentage of surviving cells relative to the DMSO group. (**C**) FTH1 mRNA expression evaluated by qRT-PCR. Statistical significance was determined using one-way ANOVA with Tukey's test. $*p < 0.05$, $****p < 0.0001$.

Figure S3

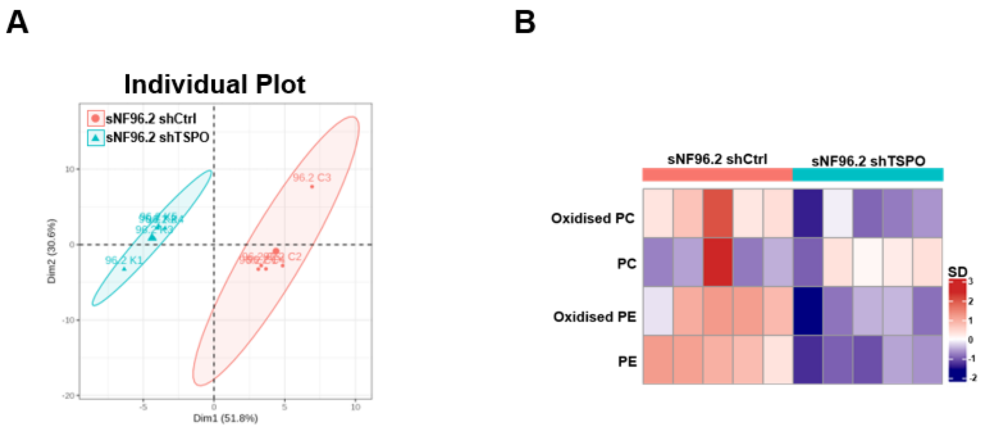

Figure S3. TSPO knockdown sNF96.2 cells exhibit distinctive oxidized phospholipid profiles.

(**A**) Principal component analysis. (**B**) Quantitative assessment of PC, oxidized PC, PE and oxidized PE molecular species in WT and TSPO knockdown sNF96.2 cells.

Figure S4

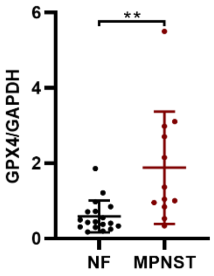

Figure S4. GPX4 mRNA expression in benign NF tissues (n=18) and malignant MPNST tissues (n=12) evaluated by RT-qPCR.

Figure S5

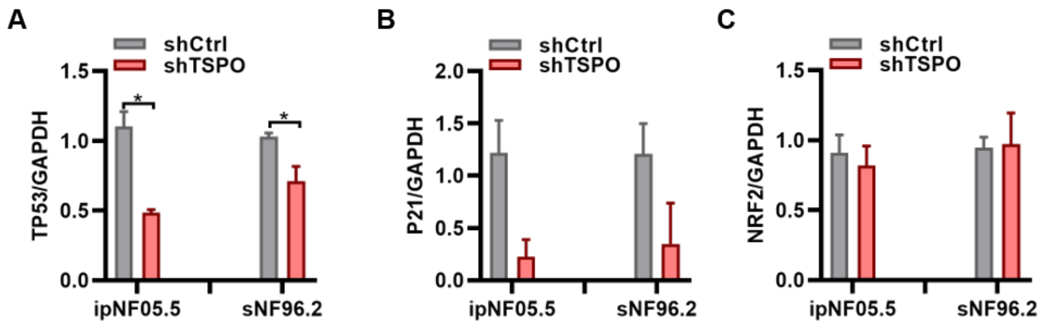

Figure S5. TP53, P21 and NRF2 mRNA expression in WT and TSPO-knockdown ipNF05.5 and sNF96.2 cells evaluated by RT-qPCR.

(A) TP53 mRNA expression. (**B**) P21 mRNA expression. (**C**) NRF2 mRNA expression. Statistical significance was determined using one-way ANOVA with Tukey's test. $*p < 0.05$.

Supplementary Table S1. Clinical information in 30 patients.

Patient	Gender	Age	Site	NF or MPNST
Patient 1#	Female	22	Rear thigh	MPNST
Patient 2#	Male	41	Pelvic cavity	MPNST
Patient 3#	Male	23	Rear thigh	MPNST
Patient 4#	Male	33	Pelvic cavity	MPNST
M5	Female	44	Cervical brachial plexus nerve	MPNST
M6	Female	45	Neck	MPNST
M7	Female	25	Neck	MPNST
M8	Female	12	Coropulum	MPNST
M9	Female	58	Forearm	MPNST
M10	Female	16	Loin	MPNST
M11	Male	54	Axillary brachial plexus nerve	MPNST
M12	Female	36	Cervical brachial plexus nerve	MPNST
NF1	Female	37	Inner thigh	NF
NF2	Male	33	Neck	NF
NF3	Female	50	Trunk	NF
NF4	Female	34	Pelvic cavity	NF
NF5	Female	28	Neck	NF
NF6	Female	17	Opisthenar	NF
NF7	Female	27	Haunch	NF
NF8	Male	50	Trunk	NF
NF9	Female	24	Forearm	NF
NF10	Male	30	Pelvic cavity	NF
NF11	Female	17	Neck	NF
NF12	Female	27	Trunk	NF
NF13	Female	33	Neck	NF
NF14	Female	26	Trunk	NF
NF15	Male	24	Inner thigh	NF
NF16	Female	64	Neck	NF
NF17	Female	10	Forearm	NF
NF18	Male	35	Neck	NF

Abbreviations

Patient 1#-4#， four malignant peripheral nerve sheath tumor patients from whom tumor tissues and adjacent non-tumor tissues were collected; M5-M12, malignant peripheral nerve sheath tumor patients; NF1-18, neurofibroma patients; MPNST, malignant peripheral nerve sheath tumor; NF, neurofibroma.

Supplementary Table S2. All primers used in the study.

Genes	Sense	Antisense
GAPDH	CTCTGCTCCTCCTGTTCGAC	GCGCCCAATACGACCAAATC
TSPO	TTCACAGAGAAGGCTGTGGTTC	GCCATACGCAGTAGTTGAGTGT
AKR1C1	TCCAGTGTCTGTAAAGCCAGG	CCAGCAGTTTTCTCTGGTTGAA
FTH1	CCAGCACCGTTTTTGTGGTT	GCCAATTCGCGGAAGAAGTG
GPX4	GAGGCAAGACCGAAGTAAACTAC	CCGAACTGGTTACACGGGAA
TP53	CAGCACATGACGGAGGTTGT	TCATCCAAATACTCCACACGC
P21	TCGTTCTCGCCCGCAATTTAG	GATAAGGACGGCTCCGTTTTG
NRF2	TCAGCGACGGAAAGAGTATGA	CCACTGGTTTCTGACTGGATGT
